# Role of miRNAs from mesenchymal stem cell–derived extracellular vesicles in neuroinflammation and behavioral impairments induced by chronic alcohol consumption in female mice

**DOI:** 10.4103/NRR.NRR-D-24-01260

**Published:** 2025-06-19

**Authors:** Susana Mellado, Najoua Touahri, Sandra Montagud-Romero, Carla Perpiñá-Clérigues, Francisco García-García, Victoria Moreno-Manzano, Consuelo Guerri, Marta Rodríguez-Arias, María Pascual

**Affiliations:** 1 Department of Physiology, School of Medicine and Dentistry, University of Valencia, Valencia, Spain; 2 Department of Psychobiology, School of Psychology, University of Valencia, Valencia, Spain; 3 Computational Biomedicine Laboratory, Príncipe Felipe Research Center, Valencia, Spain; 4 Neuronal and Tissue Regeneration Laboratory, Príncipe Felipe Research Center, Valencia, Spain

**Keywords:** behavior, chronic alcohol consumption, cognitive, ethanol, extracellular vesicles, female, mesenchymal stem cells, miRNAs, neuroinflammation

## Abstract

Mesenchymal stem cell–derived extracellular vesicles have emerged as a promising form of regenerative and immunomodulatory therapy; indeed, micro (mi)RNAs contained within mesenchymal stem cell–derived extracellular vesicles modulate target gene expression and impact disease-associated pathways. Chronic alcohol consumption leads to neuroinflammation, brain damage, and impaired cognition. Evidence indicates that females are more vulnerable to alcohol-induced damage than males. While mesenchymal stem cell–derived extracellular vesicles have been studied in various neuroinflammatory conditions, their potential to counteract alcohol-induced brain damage remains unclear. In this study, we investigated whether repeated intravenous administration of mesenchymal stem cell–derived extracellular vesicles could ameliorate neuroinflammation and behavioral impairment induced by chronic alcohol consumption in female mice. Mesenchymal stem cell–derived extracellular vesicles diminished the increased binding of a micro-positron emission tomography tracer (^18^F-FDG) when analyzing whole-brain 3D images and brain coronal sections of ethanol-treated mice. Mesenchymal stem cell–derived extracellular vesicle administration protected against ethanol-induced proinflammatory gene upregulation, cognitive dysfunction, and the conditioned rewarding effects of cocaine. MiRNA sequencing data from mesenchymal stem cell–derived extracellular vesicles revealed the elevated expression of extracellular vesicle–derived miR-483-5p and miR-140-3p in the brains of ethanol-treated female mice following mesenchymal stem cell–derived extracellular vesicle administration. In addition, mesenchymal stem cell–derived extracellular vesicles modulated the expression of pro-inflammatory-related miRNA target genes (e.g., *Socs3*, *Tnf*, *Mtor*, and *Atf6*) in the brains of ethanol-treated female mice. These results suggest that mesenchymal stem cell–derived extracellular vesicles could function as a neuroprotective therapy to ameliorate the neuroinflammation, cognitive dysfunction, and conditioned rewarding effects of cocaine associated with chronic alcohol consumption.

## Graphical abstract

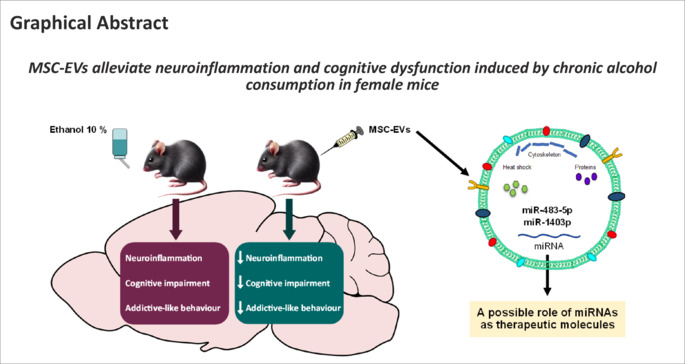

## Introduction

Mesenchymal stem cells (MSCs) have the capacity for self-renewal and multidirectional differentiation (Ye et al., 2023); however, increasing evidence suggests that their therapeutic potential derives from secreted factors, such as extracellular vesicles (EVs) (Drommelschmidt et al., 2017). MSC-EVs have garnered significant attention as promising therapeutic candidates for treating conditions like cancer, autoimmune disease, neurological disorders, and inflammation (Ye et al., 2023). Although MSC-EVs transfer numerous types of bioactive molecules (e.g., DNA, RNA, proteins, and lipids) to target cells to exert their therapeutic activity, evidence indicates that the delivery of microRNAs (miRNAs) induces the most significant impact (Xin et al., 2020). Therefore, treatment with MSC-EVs or specific miRNAs will potentially exert similar anti-inflammatory and regenerative effects as MSCs themselves (Seo et al., 2019). The use of big data–based transcriptome analysis combined with computational tools, has identified many critical factors within MSC-EVs, including functional enrichment of miRNA expression patterns and gene analysis, which has contributed to the application of MSC-EVs in targeting appropriate diseases (Seo et al., 2019).

Chronic consumption of alcohol – one of the most commonly abused substances worldwide – leads to dependence-associated alterations in brain structure and function, contributing to the development of behavioral, cognitive, and psychiatric disorders (Obad et al., 2018). Evidence indicates that females are more vulnerable to alcohol-induced damage than males, as demonstrated by a faster progression to dependence and more pronounced neurotoxic effects (Pascual et al., 2017a; Flores-Bonilla and Richardson, 2020). Additionally, intracranial gray matter is smaller in alcoholic women than in men (Erol and Karpyak, 2015). Although the underlying mechanisms of ethanol neurotoxicity remain incompletely understood, our findings reveal that ethanol induces a neuroinflammatory immune response, triggering the release of cytokines and chemokines that cause brain damage and behavioral dysfunction (Pascual et al., 2021). In the brain, glial miRNAs are transferred to neurons after alcohol consumption, leading to apoptosis and neuroinflammation (Pascual et al., 2020). Dysregulation of specific miRNAs has indeed been linked to neurodegenerative diseases and cognitive impairments associated with chronic alcohol exposure (Pascual et al., 2020; Occhipinti et al., 2023). While the administration of anti-inflammatory compounds may have potential as a short-term treatment for ethanol consumption, developing effective therapies for chronic alcohol abuse remains challenging due to the complex pathophysiology of alcohol dependence (Stokłosa et al., 2023). Our recent studies have demonstrated the potential of MSC-EVs to ameliorate the upregulation of inflammatory gene expression, NLRP3 inflammasome activation, and to recover cognitive and memory dysfunctions induced by binge drinking in adolescent mice (Mellado et al., 2023, 2025).

Considering that MSC-EVs can alleviate symptoms associated with immune disorders and neurodegenerative processes (Harrell et al., 2019; Kandeel et al., 2023), the present study aims to evaluate whether the repeated intravenous administration of EVs from human adipose tissue-derived MSCs can ameliorate the neuroinflammation, cognitive dysfunction, and the conditioned rewarding effects of cocaine induced by alcohol consumption in female mice. *In vivo* micro-positron emission tomography (microPET) studies demonstrated that MSC-EV administration reduced the increased specific binding of ^18^F-FDG in the brains of ethanol-treated female mice. MSC-EVs also protected against the ethanol-induced upregulation of proinflammatory gene expression, memory and learning impairments, and the conditioned rewarding effects of cocaine. In addition, MSC-EVs also modulated miRNAs expression and their pro-inflammatory-related miRNA target genes in the brains of ethanol-treated female mice, suggesting that the miRNAs contained within MSC-EVs play a neuroprotective role in chronic alcohol-induced neuroinflammation and behavior impairments.

## Methods

### Mesenchymal stem cell isolation and culture and mesenchymal stem cell–derived extracellular vesicle isolation

Human adipose tissue was obtained from surplus fat tissue isolated during knee prosthesis operations performed on four patients under sterile conditions. All human samples were anonymized. The experimental procedure was previously evaluated and approved by the Regional Ethics Committee for Clinical Research with Medicines and Health Products (Code of Practice 2014/01) on April 1, 2014. As exclusion criteria, no samples were collected from patients with a history of cancer or infectious (viral or bacterial) diseases. All human patients voluntarily signed an informed consent document to allow the use of the adipose samples.

MSCs were expanded, grown, and characterized, as previously described (Mellado-López et al., 2017; Muñoz-Criado et al., 2017). To collect MSC-EVs, cell media was first collected and cleared of detached cells and cell fragments by centrifugation at 300 × *g* for 10 minutes. The supernatant was then centrifuged at 2000 × *g* for 10 minutes. Subsequently, apoptotic bodies and other cellular debris were pelleted by centrifuging the supernatant at 10,000 × *g* for 30 minutes. EVs were then pelleted from the resulting supernatant at 100,000 × *g* for 1 hour. The EV pellet was washed with phosphate-buffered saline (PBS) and centrifuged at 100,000 × *g* for 1 hour. EVs were finally suspended in PBS at 20 µg/100 µL and stored at –80°C.

### Mesenchymal stem cell–derived extracellular vesicle characterization by transmission electron microscopy and nanoparticle tracking analysis

**Figure 1:**
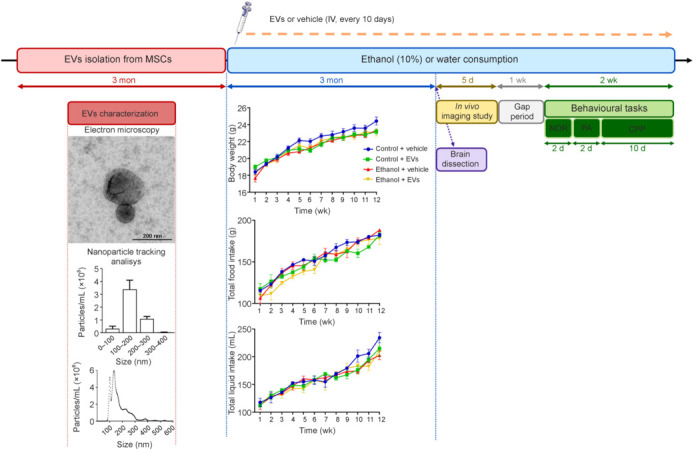
Study design. Female mice were treated with ethanol (10%) in drinking water for 3 months, while the control animals only drank water. The isolation of extracellular vesicles (EVs) from mesenchymal stem cells (MSC) took place over 3 months, during which MSC-EVs or vehicle were administered every ten days throughout the ethanol treatment period in both control and ethanol-treated mice (providing four groups: Control + vehicle, Control + EVs, Ethanol + vehicle, and Ethanol + EVs). MSC-EV characterization by nanoparticle tracking analysis revealed a peak between 100 and 200 nm, covering the size range of EVs revealed by transmission electron microscopy. Body weight, total liquid, and food intake were recorded during ethanol treatment. Values represent mean ± SEM, *n* = 12–13 mice/group. Mice at postnatal day (PND) 146 were sacrificed, and their brains were dissected. A separate group of mice (PND 146) continued receiving all treatments for an additional 5 days for *in vivo* imaging studies. Following a 1-week gap, mice at PND 160 underwent behavioral tasks, including the Novel Object Recognition (NOR) test (2 days), Passive Avoidance (PA) test (2 days), and Conditioned Place Preference (CPP) test (10 days). Throughout all experiments, the mice continued to receive both treatments.

Freshly isolated MSC-EVs were fixed with 2% paraformaldehyde and prepared as previously described (Ibáñez et al., 2019). Samples were analyzed using a transmission FEI Tecnai G2 Spirit electron microscope (FEI Europe, Eindhoven, the Netherlands) with a Morada digital camera (Olympus Soft Image Solutions GmbH, Münster, Germany). The absolute size range and concentration of EVs were measured using a NanoSight NS300 Malvern (NanoSight Ltd., Minton Park, UK), as previously described (Ibáñez et al., 2019). **Figure 1** displays a high peak in the total number of particles ranging between 100–200 nm, which includes the size range of EVs shown by transmission electron microscopy.

### Animals and treatment

Two-month-old female C57BL/6 mice weighing **~**18 g (Charles River Laboratories, Wilmington, MA, USA) were used. Mice were housed (3–4 animals/cage) and maintained on a water and solid diet ad libitum. Environmental conditions, such as light and dark cycles (12/12 hours), temperature (23°C), and humidity (60%), were controlled for all animals. All experimental procedures were carried out in accordance with the guidelines approved by the European Communities Council Directive (2010/63/ECC) and Spanish Royal Decree 53/2013 modified by Spanish Royal Decree 1386/2018, with the approval of the Ethical Committee of Animal Experimentation of the University of Valencia (Valencia, Spain) and the Generalitat Valenciana on January 20, 2023 (Project identification code: 2022 VSC PEA 0294). Female mice were selected for this study based on our previous findings, which demonstrated a heightened neuroinflammatory response in females compared to males (Alfonso-Loeches et al., 2013; Pascual et al., 2017a).

For chronic ethanol consumption, mice were treated with drinking water (control groups) or water containing 10% (v/v) ethanol for 3 months *ad libitum* as previously described (Alfonso-Loeches et al., 2010). Some animals received MSC-EVs (20 µg/dose) or saline (sodium chloride, 0.9%) in the light cycle via the tail vein every 10 days, throughout 3 months of ethanol treatment. Animals were randomly assigned to four groups according to treatment: 1) control + vehicle, 2) control + MSC-EVs, 3) ethanol + vehicle, and 4) ethanol + MSC-EVs (*n* = 23–25 mice/group). Daily food and fluid intake in the four groups were carefully measured for 3 months, with no differences observed (**Figure 1**). In addition, the body weight gained during the three months remained similar in all groups (**Figure 1**), and ethanol-treated mice are shown to be in optimal condition. From our previous study (Pascual et al., 2017b) using the same treatment schedules, similar blood ethanol levels in the dark cycle were shown in the ethanol-treated groups (125 ± 20 mg/dL), suggesting that MSC-EVs do not modify the blood ethanol levels. After ethanol treatment, some animals at postnatal day (PND) 146 were anesthetized via an intraperitoneal injection of sodium pentobarbital (60 mg/kg [200 mg/mL]; Vetoquinol, Lure, France) and then sacrificed by cervical dislocation. Brains (*n* = 8–9 mice/group) were removed, and the prefrontal cortex, striatum, and hippocampus were dissected, immediately snap-frozen in liquid nitrogen, and stored at –80°C until used. Other animals (PND 146) were scanned using an *in vivo* fluorescent imaging system, microPET, and magnetic resonance imaging (MRI) (*n* = 4–6 mice/group) and then used for behavioral studies (*n* = 13–15 mice/group) (PND 160). All behavioral tasks were performed during the dark cycle by using red light. Treatments continued throughout the entire experimental period until all procedures were completed.

### Micro-positron emission tomography experiments

MicroPET experiments were conducted after 3 months of ethanol treatment in all four groups of mice. Mice were anesthetized via 3.0%–4.0% isoflurane inhalation (Piramal Critical Care, Bethlehem, PA, USA) in 100% O_2_ and then injected intraperitoneally with 200–300 μCi of ^18^F-fluoro-2-deoxyglucose (18F-FDG; Advanced Accelerator Applications, Saint-Genis-Pouilly, France). Animals were returned to their housing cages, with 18F-FDG tracer uptake lasting approximately 30 minutes. After tracer uptake, animals were anesthetized via the inhalation of isoflurane mixed with 100% O_2_ (3.0%–4.0% for anesthetic induction and 1.0%–2.5% for anesthesia maintenance). Mice were placed on a thermo-regulated bed in the prone position and scanned with the MRS*PET CLIP-ON (MR-Solutions, Guildford, UK), which contains a continuous silicon photomultiplier detector (SiPM) with a double-layer LYSO/LYSO crystal (10 mm). For radiotracer readings (45 minutes after ^18^F-FDG injection), 15-minute list mode static acquisitions were acquired within the field of view (36.12 × 36.12 × 60.48 mm^3^) centered on the mouse head. These images were obtained using the preclinical SCAN program (MR-Solutions), calibrating time, and injected dose. All data were reconstructed using the PET reconstruction program (MR-Solutions), with a field of view diameter of 34 mm, voxel size of 0.42 mm^2^, and two iterations. During radiotracer readings, the respiratory and heart rates and body temperature were monitored and kept as constant as possible. The Paxinos mouse brain atlas (Paxinos and Franklin, 2019) and MRI and computed tomography (CT) templates were used to overlay the normalized images previously coregistered to the microPET image database. ^18^F-FDG binding was calculated in the whole brain, the prefrontal cortex, striatum (dorsal and ventral), and hippocampus (dorsal and ventral) using the VivoQuant 2022 (Invicro, Needham, MA, USA). Three-dimensional sets of regions of interest (ROI) were drawn using VivoQuant 2022 (Invicro) and then applied to individual ^18^F-FDG images to retrieve ^18^F-FDG uptake values in each brain. Therefore, the values of each ROI in each brain were divided by the whole-brain mean of the signal for ROI normalization. Data were expressed as ^18^F-FDG binding in kBq/cm^3^.

### Magnetic resonance imaging

Mice were deeply anesthetized as described for the microPET experiments. Imaging was carried out on MRS*DRYMAG 3.0T (MR-Solutions) and preclinical SCAN program (MR-Solution). T1-weighted sequences were used to visualize structural brain alterations in the whole brain, hippocampus, and cortex using MSME sequences (multi-spin multi-echo): 21.88 × 25 × 0.8 mm^3^ spatial resolution. MRI images were analyzed using ImageJ software (version 1.54h, NIH, Bethesda, MD, USA).

### *In vivo* fluorescent imaging system

IVIS® Lumina^TM^ X5 Imaging System (Revvity, Inc., Waltham, MA, USA) was used to non-invasively visualize *in vivo* brain inflammation. The IVISense^TM^ Pan Cathepsin 750 FAST Fluorescent Probe (ProSense®) (Revvity, Inc.) was used following the manufacturer’s instructions. Before the imaging session, mice received an intravenous injection of the fluorescent probe (4 nmol/mouse) dissolved in 100 µL of physiological saline. Then, mice were anesthetized with isoflurane (3.0%–4.0% for anesthetic induction and 1.0%–2.5% for anesthesia maintenance) in 100% O_2_, placed into the imaging chamber at 37°C, and imaged at various time points (0, 6, 24, 48, and 96 hours) after probe injection. The resulting light emission was quantified using the Living Image® 4.0 software (Revvity, Inc.). Data was reported as arbitrary units of radiant efficiency.

### Total RNA isolation, reverse transcription, and quantitative polymerase chain reaction

The frozen prefrontal cortex, striatum, and hippocampus were used for total RNA extraction. Tissues were disrupted using TRIzol (Sigma-Aldrich, St. Louis, MO, USA), and the total RNA fraction was extracted following the manufacturer’s instructions. Total mRNA and total miRNA were reverse-transcribed using the NZY First-Strand cDNA Synthesis Kit (NZYTech, Lda. Genes and Enzymes, Lisbon, Portugal) and TaqMan^TM^ Advanced miRNA Assays (Thermo Fisher Scientific, Waltham, MA, USA).

quantitative PCR (qPCR) was performed in a QuantStudio^TM^ 5 Real-Time PCR System (Applied Biosystems, Waltham, MA, USA). Genes were amplified employing the AceQ® qPCR SYBR Green Master Mix (NeoBiotech, Nanterre, France) following the manufacturer’s instructions. The mRNA level of the cyclophilin A housekeeping gene was used as an internal control for the normalization of analyzed genes. Specific miRNAs were amplified by the TaqMan^TM^ Fast Advanced Master Mix (Thermo Fisher Scientific), and snRNA U6 was used as an internal control. All qPCR runs included non-template controls (NTCs). Experiments were performed in triplicate. The quantification of expression (fold change) from the Cq data was calculated by the ΔΔCq method (Schmittgen and Livak, 2008) by the QuantStudio^TM^ Design & Analysis Software (Applied Biosystems). Details of the nucleotide sequences of the used primers and miRNA assays are detailed in **Additional Tables 1** and **2**).

**Additional Table 1:** Nucleotide sequences of the primers used for qPCR of genes

Gene	Primer sequences (5’ to 3’)
**Il1b**	F: GACCCCAAAAGATGAAGGGCT
	R: TGTGCTGCTGCGAGATTTGA
**Il6**	F: AAGCCAGAGTCCTTCAGAGAGA
	R: TCTTGGTCCTTAGCCACTCCT
**Ccl2**	F: AGGTCCCTGTCATGCTTCTG
	R: TCTGGACCCATTCCTTCTTG
**Ccl3**	F: AGATTCCAACGCCAATTCATC
	R: CTCAAGCCCCTGCTCTACAC
**Cx3cl1**	F: TGCGAAATCATGTGCGACAA
	R: TGGACCCATTTCTCCTTCGG
**Nos2**	F: AATCTTGGAGCGAGTTGTGG
	R: ATCTCTGCCTATCCGTCTCG
**Cox2**	F: CCGAGACAAAACAGGAGGC
	R: GGTGGGCATCTGGGAATGA
**Tnf**	F: GAACTGGCAGAAGAGGCACT
	R: AGGGTCTGGGCCATAGAACT
**Socs3**	F: CTGGTACTGAGCCGACCTCT
	R: GGCAGCTGGGTCACTTTCTC
**Mtor**	F: CTGATCCTCAACGAGCTAGTTC
	R: GGTCTTTGCAGTACTTGTCATG
**Atf6**	F: TGGAGGTCAACAAATAACACACTGG
	R: ATTCAGTCTGCTGGTTCAGTCTGG
**Cyclophilin A**	F: GTCTCCTTCGAGCTGTTTGC
	R: GATGCCAGGACCTGTATGCT
**snRNA U6**	F: GCTTCGGCAGCACATATACTAAAAT
	R: CGCTTCACGAATTTGCGTGTCAT

**Additional Table 2:** Nucleotide sequences of the primers used for qPCR of microRNAs

MicroRNA	Chromosome location	Accession number	Mature primer sequences (5’ to 3’)
mmu-miR-140-3p	Chr.16: 69933081 - 69933180 [+] on Build GRCh38	MI0000456	UACCACAGGGUAGAACCACGG
mmu-miR-483-5p	Chr.7: 142654924 - 142654996 [-] on Build GRCm38	MI0003484	AAGACGGGAGAAGAGAAGGGAG

### RNA extraction from mesenchymal stem cell-derived extracellular vesicles, miRNA sequencing, and bioinformatics analysis

#### Total mesenchymal stem cell–derived extracellular vesicle RNA isolation and library preparation

Total RNA from MSC-EVs was isolated using the Total Exosome RNA Isolation Kit, following the manufacturer’s instruction (Invitrogen, Waltham, MA, USA). The construction of miRNA libraries was performed using the Small RNA-Seq Library Prep Kit for Illumina (Lexogen, Wien, Austria). Small RNA transcripts were first converted into cDNA libraries, followed by quality and quantity assessments employing High Sensitivity DNA Chips and the 2100 Bioanalyzer System (Agilent Technologies, Santa Clara, CA, USA). The final libraries were pooled in equimolar concentration for sequencing in NextSeq 550 Sequencing System (Illumina, San Diego, CA, USA) through NextSeq 500/550 v2.5 Kit (Illumina) with 1 × 150 bp read length and 400 M maximum reads per run. The raw data generated by the high-throughput sequencing of miRNA were exported as fastq files.

#### Bioinformatics/pipelines analysis

The reads were preprocessed with Cutadapt (version 4.6, https://cutadapt.readthedocs.io/en/v4.6/; Kechin et al., 2017). After removing adapters, the trimmed sequences were aligned against the Homo sapiens sequences from miRBase mature.fa file (https://www.mirbase.org/download/; Griffiths-Jones et al., 2006) using Bowtie2 (version 2.5.3, https://bowtie-bio.sourceforge.net/bowtie2/index.shtml; Langmead and Salzberg, 2012), allowing for the detection and annotation of the mature miRNAs of interest. Following this, a count matrix of the samples was generated.

#### Functional analysis

Based on the top four most abundant miRNAs by read counts in samples that have orthologs in mice (mmu-miR-483-5p, mmu-miR-3960, mmu-miR-191-5p, mmu-miR-140-3p), the multiMiR package and database (version 1.24.0, https://bioconductor.org/packages/release/bioc/html/multiMiR.html; Ru et al., 2014) was used to obtain their validated target genes. The R version used for this analysis was 4.3.2 (https://cran.r-project.org/; R Core Team, 2021). Protein-protein interaction networks were also constructed using the STRING web tool (https://string-db.org/). Cytoscape (version 3.10.1, https://cytoscape.org/; Shannon et al., 2003), in conjunction with the Cytoscape StringApp (https://apps.cytoscape.org/apps/stringapp), was used to generate a new network based on the target genes of experimentally validated miRNAs. Proteins in the network were filtered by nervous system tissue using a threshold of 3.5, with an edge score set at 0.6, thereby reducing the number of target genes for subsequent functional enrichment analysis. Functional enrichment analysis was performed using pathways from the KEGG (Kyoto Encyclopedia of Genes and Genomes) (https://www.genome.jp/kegg/), Reactome (https://reactome.org/), and WikiPathways databases (https://www.wikipathways.org/).

### Behavioral testing

#### Novel object recognition test

The novel object recognition test assesses cognitive function by measuring the ability to recognize and explore new objects in a neutral environment, free of aversive or reinforcing stimuli (Ennaceur and Delacour, 1988; Lueptow, 2017). Mice (PND 160) performed the novel object recognition test in a black open box (24 cm × 24 cm × 15 cm) using small, non-toxic objects: two plastic boxes and a plastic toy. According to a study by Mellado et al. (2023), the task procedure consists of three phases: habituation, the training session (T1), and the test session (T2). During the habituation session, mice spent 5 min exploring the open-field arena where T1 and T2 were performed. During the training session, one mouse was placed in the open-field arena containing two identical sample objects placed in the middle of the testing box for 3 minutes. After a 1-minute retention interval, the animal was returned to the open-field arena with two objects during the test session (3 minutes): one object was identical to the sample, and the other was novel. The objects size for the T1 we employed two similar river stones 3 × 2.5 × 0.5 cm^3^ (length × width × height). For the novel objects (a small toy), the size was 2 × 1 × 4 cm^3^. The recognition index was calculated by measuring the discrimination index (%) = (tnovel – tfamiliar)/(tnovel + tfamiliar) × 100, with “t” taken as the time that each mouse spent exploring an object. In addition, the investigation time in the T1 and T2 were scored and is showed in **Additional Figure 1**.

#### Passive avoidance test

The passive avoidance task measures the latency to enter in a dark compartment, in which an aversive stimulus (foot shock) has been previously experienced (Sanei and Saberi-Demneh, 2019). This test employed a step-through inhibitory avoidance apparatus for mice (Ugo Basile, Comerio-Varese, Italy). This cage was made of Perspex sheets and divided into two compartments (15 × 9.5 × 16.5 cm^3^ [length × width × height]). The safe compartment was white and illuminated by a light fixture (10 W) fastened to the cage lid, whereas the “shock” compartment was dark and made of black Perspex panels. Both compartments were divided by a door that was automatically operated by sliding on the floor. The floor was made of 48 stainless steel bars (0.7 mm in diameter) placed 8 mm apart. Passive avoidance tests were carried out following the procedure previously described in mice at PND 162 (Mellado et al., 2023).

#### Conditioned place preference

The conditioned place preference test is a widely used behavioral paradigm to assess the rewarding effects of drugs and the ability to form associative memories between environmental cues and drug exposure (Hoffman, 1989). Eight identical Plexiglas boxes with two compartments of equal size (30.7 × 31.5 × 34.5 cm^3^ [length × width × height]) separated by a gray central area (13.8 × 31.5 × 34.5 cm^3^ [length × width × height]) were employed for place conditioning in mice at PND 164. The compartments had different colored walls (black *vs.* white) and distinct floor textures (fine grid in the black compartment *vs.* wide grid in the white compartment). Four infrared light beams in each compartment of the box and six in the central area allowed the position of the mice and their crossings from one compartment to the other to be recorded. The equipment was controlled by three computers using MONPRE 2Z software (Cibertec, Madrid, Spain). Place conditioning, consisting of three phases, was carried out during the dark cycle following an unbiased procedure regarding initial spontaneous preference (Manzanedo et al., 2001).

During the first phase (pre-conditioning [Pre-C]), mice were allowed access to both apparatus compartments for 900 seconds per day on three consecutive days. On day 3, the time spent in the associated and non-associated compartment, as well as the total number of entries or crossings during the Pre-C phase was recorded (**Additional Tables 3** and **4**, respectively). Mice showing a strong unconditioned aversion (< 33% of session time; i.e., 250 seconds) or preference (> 67 % of the session time; i.e., 650 seconds) for any compartment were discarded from the rest of the study (a total of six animals were excluded from the test after the pre-conditioning phase: one animal from the control group, two from the ethanol group, two from the MSC-EV-treated group, and one from the ethanol + MSC-EV-treated group). No significant differences between the time spent in the drug-paired and vehicle-paired compartments during the Pre-C phase were found.

**Additional Table 3:** Time spent (in seconds) in the non-associated compartment and associated compartment in both pre-conditioning and post-conditioning sessions in the conditioned place preference task

Experimental group	Non-associated compartment	Associated compartment
***Pre-conditioning session***	390 ± 20.59 (*n* = 14)	360 ± 21.14 (*n* =14)
Control	406 ± 24.95 (*n* = 13)	364 ± 18.31 (*n* =13)
Ethanol	369 ±21.50 (*n* =13)	375 ± 16.88 (*n* =13)
EVs	391 ±23.00 (*n* =13)	384 ± 18.62 (*n* =13)
EVs + Ethanol		
***Post-conditioning session***	331 ±23.40 (*n* =14)	406 ± 24.26 (*n* = 14)
Control	345 ± 17.68 (*n* =13)	380 ± 19.21 (*n* =13)
Ethanol	297 ±23.40 (*n* =13)	418 ± 24.26 (*n* =13)
EVs		
EVs + Ethanol	313 ± 17.68 (*n* =13)	409 ± 19.21 (*n* =13)

Data represent mean ± SEM. (1) The time spent in the non-associated compartment decreased in the Post-C test (*P* < 0.001), but the time spent in the associated compartment increased significantly (*P* < 0.05). These results are expected because three groups developed preference for cocaine. (2) In the Post-C test, the time spent in all groups in the associated compartment is significantly higher than the time spent in the non-associated compartment. EVs: Extracellular vesicles; post-C: Post-conditioning.

**Additional Table 4:** The total number of entries or crossings during the post-conditioning sessions in the conditioned place preference task

Experimental group	
***Pre-conditioning session***	
Control	51 ± 2.6 (*n* =14)
Ethanol	44 ± 3.5 (*n* =13)
EVs	46 ± 3.5 (*n* =13)
EVs + Ethanol	49 ± 3.2 (*n* =13)
***Post-conditioning session***	
Control	80 ± 5.6 (*n* =14)
Ethanol	81 ± 4.1 (*n* =12)
EVs	82 ± 6.7 (*n* =13)
EVs + Ethanol	82 ± 5.5 (*n* =13)

Data represent mean ± SEM. EVs: Extracellular vesicles.

In the second phase (conditioning), which lasted 4 days, mice were conditioned with 1.5 mg/kg cocaine hydrochloride (Alcaliber laboratory, Madrid, Spain) or physiological saline. The cocaine dose was selected based on previous conditioned place preference studies showing that doses below 3 mg/kg are subthreshold (Arenas et al., 2014; Montagud-Romero et al., 2017; Reguilón et al., 2022). During this phase, half of the mice in each group received the drug or vehicle in one compartment, while the other half received it in the other compartment. An injection of physiological saline was administered before confining mice to the vehicle-paired compartment for 30 minutes. After an interval of 4 hours, the mice received cocaine immediately before confinement in the drug-paired compartment for a further 30 minutes. The central area was made inaccessible by guillotine doors during conditioning. The dose of cocaine used during the conditioning phase was a subthreshold dose (1.5 mg/kg, proven to be ineffective in controls) to evaluate increased sensitivity to the conditioned rewarding effects of cocaine.

In the third phase (postconditioning [Post-C]), which took place on day eight, the guillotine doors separating the two compartments were removed, and the time spent in each compartment by the untreated mice during a 900-second observation period was recorded. The difference in seconds between the time spent in the drug-paired compartment during the Post-C test and the Pre-C phase measures the degree of conditioning induced by the drug. A positive difference suggests that the drug has induced a preference for the drug-paired compartment, while a negative difference indicates the development of an aversion. In addition, the time spent in the associated and non-associated compartment, as well as the total number of entries or crossings during the Post-C phase was recorded (**Additional Tables 3** and **4**).

For extinction, mice were placed in the conditioned place preference apparatus daily, and the time spent in each compartment was measured to determine if cocaine-induced preference had disappeared. Although the group’s mean determined the day extinction was considered to have been achieved, preference was considered extinguished when a mouse spent 378 seconds or less in the drug-paired compartment on 2 consecutive days. This time was chosen based on the values of all the Pre-C tests performed in the study (mean = 360 seconds). When the preference was not extinguished in an animal, the number of days required for extinction for the group as a whole was assigned. Finally, mice were challenged with a cocaine injection once 24 hours after reaching the extinction criterion, followed by a place preference test (reinstatement test). Priming injections were administered in the vivarium, which constituted a non-contingent place to the previous conditioning procedure.

#### Statistical analysis

G*Power (version 3.1, https://www.psychologie.hhu.de/arbeitsgruppen/allgemeine-psychologie-und-arbeitspsychologie/gpower) was used to predetermine sample sizes. The results are reported as mean ± SEM. All statistical data analysis was performed using SPSS Statistics v28 (IBM Corp, Armonk, NY, USA). The Shapiro-Wilk test was used to analyze data distribution normality. A two-way analysis of variance (ANOVA) was used with two between-subjects variables: EVs (vehicle *vs.* EVs) and ethanol (control *vs.* ethanol), followed by Tukey’s *post hoc* comparisons test for *in vivo* imaging studies and qPCR. Values of *P* < 0.05 were considered statistically significant. Behavioral data from the novel object recognition and the open field were analyzed by a two-way ANOVA with two between-subject variables: ethanol (saline and ethanol); MSC-EVs (with and without EVs). Passive avoidance was analyzed with the Kruskal-Wallis tests and for the pairwise comparisons the Mann-Whitney *U* test was performed. To evaluate CPP acquisition, the difference between the time spent in the drug-paired compartment during the post- and the pre-C phases was analyzed with a *t*-test. In addition, to evaluate (in the CPP) the number of entries during the Post-C test, and for the time spent in the drug-associated and non-associated compartment during the Pre- and Post-C test, a two-way ANOVA with three between-subjects’ variables ethanol (saline and ethanol); MSC-EVs (with and without EVs); Compartment (associated and non-associated); and one within subjects’ variable – Test (Pre-C and Post-C session). Bonferroni adjustment was employed for the post hoc comparisons in the ANOVA. A detailed summary of all statistical results, including *F*-values, degrees of freedom and *p* values for each comparison has been provided in **Additional Table 5.**

## Results

### Micro-positron emission tomography imaging reveals that mesenchymal stem cell–derived extracellular vesicles ameliorate ethanol-induced neuroinflammation

**Figure 2:**
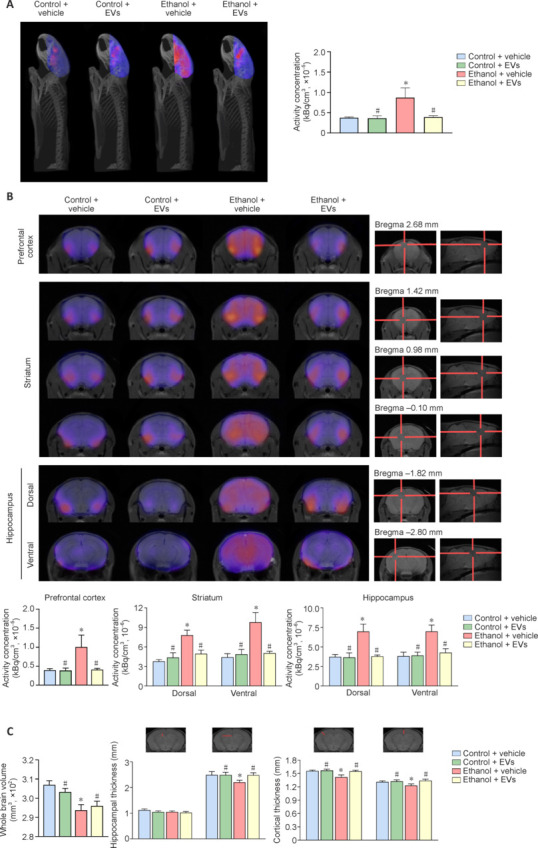
MicroPET analysis reveals that MSC-EVs decrease ethanol-induced brain ^18^F-FDG uptake in mice. (A) Representative three-dimensional images (left) and quantification of ^18^F-FDG uptake (right) in the brains of mice treated with ethanol and/or MSC-EVs for 3 months (and controls). Data represent mean ± SEM, *n* = 5–6 mice/group. (B) Representative coronal images (top, left) and quantification of ^18^F-FDG uptake (bottom) in the prefrontal cortex, striatum (dorsal and ventral), and hippocampus (dorsal and ventral) of mice treated with ethanol and/or MSC-EVs for 3 months (and controls). Coronal and sagittal MRI templates used for coregistration in the prefrontal cortex, striatum, and hippocampus (top, right). Data represent mean ± SEM, *n* = 4–6 mice/group. (C) Representative coronal MRI images and quantification of hippocampal and cortical thickness of mice treated with ethanol and/or MSC-EVs for 3 months (and controls). Data represent mean ± SEM, *n* = 5–6 mice/group. *P < 0.05, *vs.* control + vehicle-treated mice; #*P* < 0.05, *vs*. ethanol + vehicle-treated mice. Two-way analysis of variance with Tukey multiple comparisons test was performed. ^18^F-FDG: Micro-positron emission tomography tracer; CPP: conditioned place preference; MSC-EVs: extracellular vesicles derived from mesenchymal stem cells; NOR: novel object recognition; PA: passive avoidance test.

Our previous results demonstrated the therapeutic role of MSC-EVs in ameliorating neuroinflammation induced by binge ethanol drinking in adolescent mice (Mellado et al., 2023, 2025). To explore the potential protective effects of MSC-EVs in chronic alcohol consumption, we first investigated whether MSC-EVs could reduce the neuroinflammation induced by chronic ethanol exposure in our experimental model using *in vivo* microPET imaging. The glucose analog ^18^F-FDG identifies localized metabolic alterations and is a well-established imaging tracer for the differential diagnosis of neuroinflammatory processes (Palandira et al., 2022) and neurodegenerative diseases (Bouter and Bouter, 2019). **Figure 2A** shows a set of 3D images obtained by microPET, depicting the whole brains of mice in the different experimental groups. Averaged images of ^18^F-FDG uptake revealed significantly higher tracer binding in ethanol-treated mice compared with the control group (*P* < 0.05). However, MSC-EV administration in ethanol-treated animals diminished the increased ^18^F-FDG uptake induced by chronic ethanol consumption (*P* < 0.05), bringing it to a level similar to the control group (**Figure 2A**, right).

Further microPET analysis mapped the ^18^F-FDG distribution across distinct brain regions - the prefrontal cortex, striatum (dorsal and ventral), and hippocampus (dorsal and ventral) - which are involved in cognition, risk/reward, motivation, and addictive-like behavior (Bast et al., 2017; Lee et al., 2019; Chen et al., 2020). Specifically, the dorsal striatum regulates cognition and movement, while the ventral striatum modulates reward and emotion (Chen et al., 2020). The dorsal hippocampus is involved in spatial processing, whereas the ventral hippocampus processes anxiety-based behaviors (Lee et al., 2019). We observed significantly higher ^18^F-FDG binding in these regions when analyzing coronal brain sections of ethanol-treated mice compared to controls (*P* < 0.05; **Figure 2B**). Furthermore, MSC-EVs administration in ethanol-treated mice significantly reduced the tracer binding to levels similar to those of the control group.

Additionally, we evaluated structural changes in the whole brain, hippocampus, and cortex using MRI (**Figure 2C**; top – representative images). The acquired images clearly defined the anatomic boundaries of these regions (Scharwächter et al., 2022). Ethanol treatment significantly reduced whole brain volume (*P* < 0.05), hippocampal thickness (*P* < 0.05), and cortical thickness (*P* < 0.05) compared to controls. However, MSC-EV administration in ethanol-treated mice restored these ethanol-induced structural alterations to levels comparable to the control group (**Figure 2C**; bottom–quantification). Of note, no significant differences were observed between control and MSC-EV-treated animals in the microPET and MRI data (**Figure 2A–C**).

Overall, these findings indicate that MSC-EVs mitigate both functional and structural brain alterations associated with chronic ethanol exposure, supporting their potential as a neuroprotective therapy.

### Mesenchymal stem cell–derived extracellular vesicles reduce inflammatory gene expression in the prefrontal cortex, striatum, and hippocampus of ethanol-treated mice

**Figure 3:**
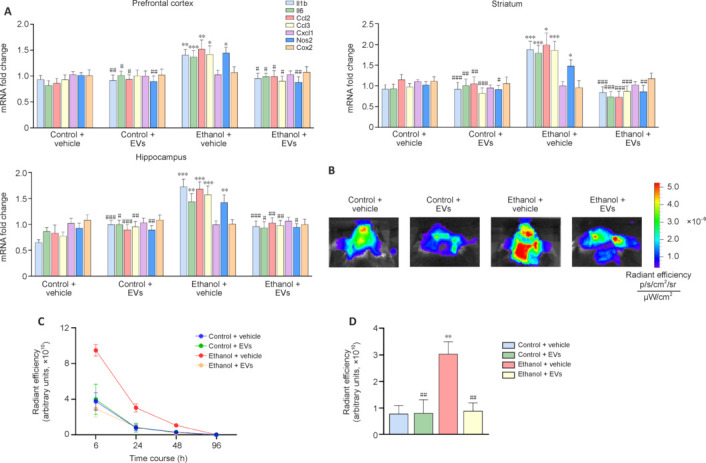
MSC-EVs decrease ethanol-induced inflammatory gene expression and *in vivo* neuroinflammation in mice. (A) mRNA levels of Il1b, Il6, Ccl2, Ccl3, Cx3cl1, Nos, and Cox2 analyzed in the prefrontal cortex, striatum, and hippocampus of mice treated with ethanol and/or MSC-EVs for three months (and controls). Data represent mean ± SEM, *n* = 8–9 mice/group. (B) Representative *in vivo* fluorescent imaging of neuroinflammation in mice treated with ethanol and/or MSC-EVs for 3 months (and controls). (C) Time-course displays the fluorescent intensity at 6, 24, 48, and 96 hours post-injection. (D) Fluorescence quantification at 24 hours after injection of the fluorescent probe. Data represent mean ± SEM, *n* = 4–6 mice/group. *P < 0.05, **P < 0.01, ***P < 0.001, *vs*. control + vehicle-treated mice; #*P* < 0.05, ##*P* < 0.01, ###*P* < 0.001, *vs*. ethanol + vehicle-treated treated mice. Two-way analysis of variance with Tukey multiple comparisons test was performed. MSC-EVs: Extracellular vesicles derived from mesenchymal stem cells.

To further confirm the neuroprotective role of MSC-EVs in ethanol-treated mice, we assessed their impact on neuroinflammation at the molecular level by analyzing inflammation-associated gene expression in key brain regions. We measured the expression levels of Il1b, Il6, Ccl2, Ccl3, Cx3cl1, Nos2, and Cox2 in the prefrontal cortex, striatum, and hippocampus. **Figure 3A** shows that ethanol treatment significantly upregulated the expression of Il1b, Il6, Ccl2, Ccl3, and Nos2 in the prefrontal cortex (Il1b, *P* < 0.01; Il6, *P* < 0.001; Ccl2, *P* < 0.01; Ccl3, *P* < 0.05; Nos2, *P* < 0.05), striatum (Il1b, *P* < 0.001; Il6, *P* < 0.001; Ccl2, *P* < 0.05; Ccl3, *P* < 0.001; Nos2, *P* < 0.05), and hippocampus (Il1b, *P* < 0.001; Il6, *P* < 0.01; Ccl2, *P* < 0.001; Ccl3, *P* < 0.001; Nos2, *P* < 0.01) compared to control. No significant differences were observed in the expression of these genes between MSC-EV-treated and control animals in any brain region. However, MSC-EV administration attenuated the ethanol-induced increase in inflammation-associated gene expression in the prefrontal cortex, striatum, and hippocampus.

Next, we employed a non-invasive *in vivo* imaging system to monitor neuroinflammation using a fluorescent probe that measures lysosomal cathepsin activity, a marker of inflammatory-related processes (Yadati et al., 2020). **Figure 3B** depicts representative examples of brain imaging with the fluorescent probe, and **Figure 3C** provides the quantification at 6, 24, and 48 hours post-injection. The absence of a fluorescent signal at 96 hours post-injection confirmed probe elimination from living mice. Fluorescent intensity significantly increased at 24 hours post-injection in ethanol-treated mice compared to control (*P* < 0.05; **Figure 3D**). Notably, MSC-EV administration at 24 hours reduced the fluorescent signals in ethanol-treated animals.

These findings indicate that MSC-EVs restore the inflammatory immune response in the prefrontal cortex, striatum, and hippocampus.

### Mesenchymal stem cell–derived extracellular vesicles restore ethanol-induced behavioral impairments

We assessed whether the MSC-EV-induced amelioration of ethanol-induced neuroinflammation was sufficient to restore cognitive dysfunction and conditioned reward learning processes in mice by performing several behavioral tasks, including novel object recognition, passive avoidance, and conditioned place preference. The novel object recognition test evaluates cognitive abilities to approach and explore novel objects in the absence of aversive or motivational stimuli (Ennaceur and Delacour, 1988; Lueptow, 2017), while the passive avoidance paradigm is commonly used to explore short- and long-term memory (Sanei and Saberi-Demneh, 2019).

**Figure 4:**
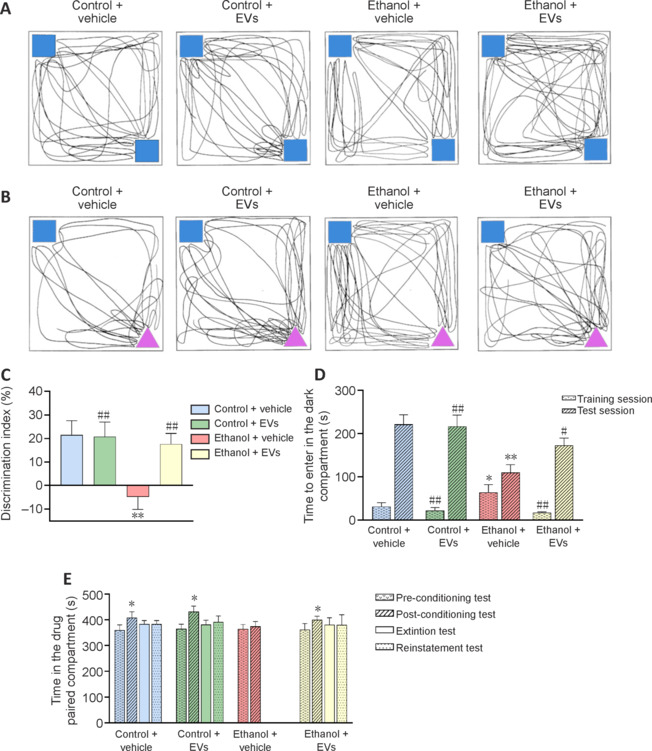
MSC-EVs restore ethanol-induced behavioral impairments in mice. (A, B) Representative trajectories of a single mouse from each experimental group of mice treated with ethanol or water and MSC-EVs or vehicle in an open box equipped with two familiar objects (represented by blue squares) (A) and familiar (represented by a blue square) and novel (represented by a pink triangle) objects (B). (C) Bar graphs represent the discrimination index during the novel object recognition task. Data are presented as mean ± SEM, *n* = 14–15 mice/group. **P < 0.01, *vs.* control + vehicle-treated mice; ##*P* < 0.01, *vs.* ethanol + vehicle-treated mice. Two-way analysis of variance with Bonferroni multiple comparisons test was performed. (D) Bar graphs represent the time taken to enter the dark compartment of the passive avoidance test during the training and test session (24 hours after training). Data are presented as mean ± SEM, *n* = 13–15 mice/group. *P < 0.05, **P < 0.01, *vs*. control + vehicle-treated mice; #*P* < 0.05, ##*P* < 0.01, *vs.* ethanol + vehicle-treated mice. Kruskal-Wallis test with Mann-Whitney *U* *post hoc* tests was performed. (E) Bar graphs represent the time spent in the drug-paired compartment before the conditioning test (Pre-C), after the conditioning test (Post-C), and in the extinction and reinstatement tests induced by a cocaine dose of 1.5 mg/kg. Data are presented as mean ± SEM, *n* = 13–14 mice/group. *P < 0.05, *vs*. Pre-C test. Two-way analysis of variance with Bonferroni multiple comparisons test was performed. MSC-EVs: Extracellular vesicles derived from mesenchymal stem cells.

A representative trajectory of the novel object recognition test, shown in **Figure 4A**, demonstrates that all groups displayed similar exploration trajectories while interacting with both familiar objects in the training session. However, ethanol-treated mice spent more time exploring the familiar objects than the other experimental groups (**Additional Figure 1**). During the test session, control, MSC-EV-treated, and ethanol + MSC-EV-treated mice spent more time exploring the novel object compared to the familiar object, while ethanol-treated mice showed higher trajectories close to the familiar object than the novel object (**Figure 4B**). In addition, ethanol-treated mice spent more time exploring both the novel and familiar objects compared to the other groups (**Additional Figure 1**). The discrimination index, used to assess novelty recognition, revealed a significant reduction in the index value in ethanol-treated mice compared to all other groups (*P* < 0.01; **Figure 4C**).

In the passive avoidance test (**Figure 4D**), ethanol-treated mice took longer to enter the dark compartment during the training session than mice from other groups (*P* < 0.05 *vs.* control + vehicle; *P* < 0.01 *vs.* control + EVs and ethanol + EVs). Moreover, during the 24-hour test, mice from all groups displayed longer step-through latency to enter the dark compartment compared to the training day; however, ethanol-treated mice displayed significantly shorter latency than the other experimental groups (*P* < 0.05 *vs*. ethanol + EVs; *P* < 0.01 *vs.* control + vehicle and control + EVs) (**Figure 4D**).

In the conditioned place preference test (**Figure 4E**), we analyzed the time spent in the drug-paired compartment after a low dose of cocaine (1.5 mg/kg). The results showed a significant increase in the time spent in the drug-paired compartment during the post-conditioned test compared to the pre-conditioned test in the control (*P* < 0.05), MSC-EV-treated (*P* < 0.01), and ethanol + MSC-EV-treated mice (*P* < 0.01). However, no significant effect was observed in ethanol-treated mice, indicating that these animals displayed reduced sensitivity to the low cocaine dose used in the conditioned place preference test.

These behavioral findings suggest that MSC-EV administration counteracts ethanol-induced cognitive impairment and protects against ethanol-modulated conditioned rewarding effects of cocaine.

### miRNA sequencing revealed potential key targets of mesenchymal stem cell–derived extracellular vesicles

**Figure 5:**
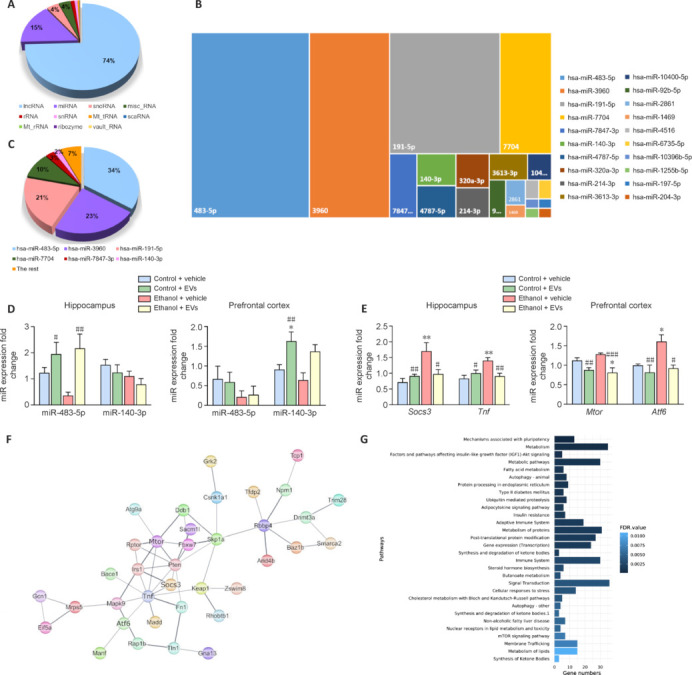
miRNA sequencing reveals potential key targets in MSC-EVs. (A) Abundance of different RNA biotypes in MSC-EVs. (B) The top 20 miRNAs in MSC-EVs. (C) Percentage of the abundance of the top six miRNAs in MSC-EVs. (D) The levels of miR-483-5p and miR-140-3p in the prefrontal cortex and hippocampus of mice treated with ethanol and/or MSC-EVs for 3 months (and controls). Data are expressed as the mean ± SEM, *n* = 6–7 mice/group. *P < 0.05, *vs*. control + vehicle mice; #*P* < 0.05, ##*P* < 0.01, *vs*. ethanol + vehicle treated mice. Two-way analysis of variance with Tukey multiple comparisons test was performed. (E) Expression levels of miR-483-5p target genes in the hippocampus (*Socs3* and *Tnf*) and miR-140-3p target genes in the prefrontal cortex (*Mtor* and *Atf6*) of mice treated with ethanol and/or MSC-EVs for 3 months (and controls). Data are expressed as the mean ± SEM, *n* = 6–7 mice/group. *P < 0.05, **P < 0.01, *vs.* control mice; #*P* < 0.05, ##*P* < 0.01, ###*P* < 0.001, *vs*. ethanol-treated mice. Two-way analysis of variance with Tukey multiple comparisons test was performed. (F) The protein–protein interaction network for the predicted miR-483-5p and miR-140-3p target genes was filtered based on nervous system tissue, and increased edge score. Validated target genes are highlighted in bold (*Mtor*, *Atf6*, *Socs3*, and *Tnf*), and line thickness represents the strength of data support. (G) The bar plot displays the top 30 most significant enriched pathways within the genes from the previous protein-protein interaction network. The bar size indicates the number of genes involved in each pathway, while the color indicates the FDR value. MSC-EVs: Extracellular vesicles derived from mesenchymal stem cells.

To explore the possible molecular mechanisms underlying the therapeutic effects of MSC-EVs, we performed high-throughput sequencing to identify miRNA species and their abundance in MSC-EVs. According to the sequencing data, miRNAs comprised the second-largest proportion of total RNA biotypes in MSC-EVs (**Figure 5A**). **Figure 5B** reveals the top 20 mature miRNAs by abundance, with miR-483-5p, miR-3960, miR-191-5p, miR-7704, miR-7847-3p, and miR-140-3p constituting nearly 87% of the total miRNA content in MSC-EVs (**Figure 5C**).

Next, we identified the orthologous mouse miRNAs corresponding to the six most abundant human miRNAs and focused on evaluating the roles of miR-483-5p and miR-140-3p in mitigating inflammatory processes (Stengel et al., 2020; Zhu et al., 2021; Wang et al., 2022; Zhou et al., 2022). We analyzed the expression of miR-483-5p and miR-140-3p in the prefrontal cortex and hippocampus of our experimental groups. Notably, the administration of MSC-EVs significantly increased the levels of miR-483-5p in the hippocampus of ethanol-treated mice compared to those that were only ethanol-treated (*P* < 0.01; **Figure 5D**).

To evaluate whether these miRNAs mediate the neuroprotective effects of MSC-EVs, we performed qPCR to assess the expression of some inflammatory target genes of miR-483-5p (e.g., *Socs3* and *Tnf*) (Zhu et al., 2021; Zhou et al., 2022) and miR-140-3p (e.g., *Mtor* and *Atf6*; Stengel et al., 2020; Wang et al., 2022). These genes were identified through a functional protein association network (**Additional Figure 2**). Ethanol treatment significantly upregulated the expression of *Mtor* and *Atf6* in the prefrontal cortex (*P* < 0.05) and *Socs3* and *Tnf* in the hippocampus compared to control (*P* < 0.01; **Figure 5E**). Moreover, MSC-EVs administration to ethanol-treated mice significantly decreased the expression of these target genes (*Tnf*, *P* < 0.05; *Mtor*, *P* = 0.001) suggesting that the neuroprotective effects of MSC-EVs may be mediated by miR-483-5p and miR-140-3p and their target genes. The protein-protein interaction network derived from these target genes, filtered by nervous system tissue expression, included the validated genes *Socs3*, *Tnf*, *Mtor*, and *Atf6* (**Figure 5F**). Functional enrichment analysis of this network revealed pathways related to autophagy, cellular response to stress, and the immune system (**Figure 5G**). Key pathways identified included Toll-like receptor signaling, the MyD88 cascade, and the MAPK signaling pathway, among others (**Additional Figure 3**).

These findings highlight a potential molecular mechanism by which MSC-EVs exert their neuroprotective effects, supporting the role of EV-derived miRNAs in modulating inflammation-related pathways and restoring brain homeostasis after chronic ethanol exposure.

## Discussion

Various studies have highlighted the regenerative potential of MSC-EVs in combating neuroinflammation, brain damage, and neurodegenerative processes (Harrell et al., 2019; Kandeel et al., 2023). Here, we provide evidence for the neuroprotective role of MSC-EVs in mouse neuroinflammation, specifically with regard to the amelioration of ethanol-induced alterations to *in vivo* microPET imaging, proinflammatory gene expression, and behavior, which could involve the transfer of miRNAs resident in MSC-EVs. Taken together, these results confirm our previous findings associated with the therapeutic function of MSC-EVs in a mouse model of binge ethanol drinking during adolescence (Mellado et al., 2023, 2025).

Alcohol consumption prompts brain damage and impaired cognitive functioning, with human and animal studies supporting the role of the neuroimmune system in the effects of ethanol on the central nervous system (Montesinos et al., 2016; Squillace and Salvemini, 2022). Our previous studies demonstrated that by activating the innate immune receptor Toll-like receptor 4 in glial cells, ethanol induces the release of cytokines and chemokines, causing neuroinflammation and neural damage (Fernandez-Lizarbe et al., 2009; Alfonso-Loeches et al., 2010). In this study, we showed that chronic ethanol consumption upregulated inflammatory gene expression (e.g., *Il1b*, *Il6*, *Ccl2*, *Ccl3*, and *Nos2*), which correlates with increased ^18^F-FDG uptake in the prefrontal cortex, striatum (dorsal and ventral), and hippocampus (dorsal and ventral). The ^18^F-FDG radiotracer is used in diagnosing and treating various disorders, including inflammatory and neuroinflammatory diseases, providing insights into neuronal and glial inflammation (Palandira et al., 2022). For instance, studies have reported enhanced ^18^F-FDG microPET signals in mouse models of Alzheimer’s disease (Jeong et al., 2017; Choi et al., 2021) and murine neuroinflammatory conditions such as amyloidosis (Xiang et al., 2021).

Part of the regenerative benefit from stem cell therapy may arise through secreted EVs, generating significance in their clinical applications. EVs from various cell sources, including MSCs, have shown efficacy in models of neurological diseases such as Alzheimer’s disease, Parkinson’s disease, and multiple sclerosis (Reed and Escayg, 2021). In these models, EVs attenuate proinflammatory signaling brain damage, and reduce cognitive and behavioral deficits (Reed and Escayg, 2021). Consistent with these studies, we demonstrated that intravenous MSC-EVs administration prevents the ethanol-induced upregulation of brain radiotracer signal in microPET, MRI structural alterations, and neuroinflammatory gene expression in adult mice. Moreover, the therapeutic effects of MSC-EVs on neuroinflammatory responses may correlate with the restoration of spatiotemporal memory dysfunction and recognition memory deficits, as assessed by the novel object recognition and passive avoidance tasks. Similarly, administration of neural stem cell-derived EVs can alleviate lipopolysaccharide-induced chronic neuroinflammation and cognitive impairments (Ayyubova et al., 2023). A previous study has also shown that MSC-EVs inhibit the chronic activation of NLRP3 inflammasome signaling and prevent long-term cognitive dysfunction following traumatic brain injury (Kodali et al., 2023). In Alzheimer’s disease (Liu et al., 2022) and status epilepticus (Long et al., 2017), MSC-EVs have also been shown to improve cognitive and behavioral impairments by regulating hippocampal inflammation.

While the therapeutic role of MSC-EVs is well-established in the context of cognitive dysfunction, their involvement in conditioned reward learning processes remains unexplored. Interestingly, our results demonstrated that miRNAs encapsulated within MSC-EVs (and their target genes) protect against ethanol-modulated conditioned rewarding effects of cocaine. Ethanol-treated mice exhibited lower sensitivity to a low dose of cocaine compared to the other experimental groups. These mice required a higher dose of cocaine to reproduce similar effects to those observed in the ethanol + MSC-EVs-treated animals, suggesting that the miRNAs contained in MSC-EVs play a role in the reward system. Likewise, some studies have shown that EV miRNAs exert protective effects against methamphetamine dependence (Zhou et al., 2021), and the use of lentiviral vector-miRNA silencers abolished cocaine-induced conditioned place preference (Chandrasekar and Dreyer, 2011). A recent study also reported that implicit cognition plays a vital role in addiction development and maintenance (Kessling et al., 2023). These cognitive processes toward addiction-related stimuli develop through conditioning and are linked to cue reactivity and craving (Kessling et al., 2023). In this sense, MSC-EVs could participate in mechanisms such as neuroinflammation and myelin and synaptic alterations to repair memory and learning impairments (Mellado et al., 2023), potentially contributing to the reinforcing properties of abused drugs.

The conditioned place preference paradigm assesses the conditioned rewarding effects of addictive substances by pairing drug effects with previously neutral cues, such as a specific compartment of an apparatus. Contextual stimuli can thus acquire secondary appetitive properties when associated with cocaine, indicating abuse potential (Tzschentke, 2007). Prolonged ethanol use downregulates the reward system, reducing dopamine release and receptor sensitivity (Koob and Le Moal, 2001). The dopaminergic system remains crucial for cocaine’s psychomotor stimulant and reinforcing effects, as demonstrated through intravenous self-administration and conditioned place preference studies (Caine et al., 2007; Montagud-Romero et al., 2016). Consequently, we hypothesize that ethanol-treated mice fail to associate a low dose of cocaine with a specific compartment due to reward system downregulation, while MSC-EVs may reverse these ethanol-induced alterations, restoring cocaine-induced preference.

The evidence that MSC-EV miRNAs play a role in overall therapeutic effects is compelling (Seo et al., 2019). By combining miRNA transcriptomics and functional bioinformatics, the potential neuroprotective implications of EV-derived miRNAs can be addressed in relation to target disease (Seo et al., 2019). Using miRNA sequencing, we identified the cargo carried by MSC-EVs and found that miR-483-5p in the hippocampus and miR-140-3p in the prefrontal cortex were more highly expressed in ethanol + MSC-EV-treated mice than in ethanol-treated mice. Both miRNAs ranked among the top six miRNAs by proportion, constituting 87% of all mature miRNAs. Previous research have shown that exosomal miR-483-5p derived from adipose stem cells exhibits anti-inflammatory properties by modulating the NLRP3 inflammasome in deep vein thrombosis (Fan et al., 2024). Similarly, MSC-derived exosome-mediated delivery of miR-140-3p alleviated hippocampal inflammatory responses, pyroptosis, and cognitive impairment in mice with sepsis-associated encephalopathy (Ma et al., 2024). Our results demonstrate that MSC-EVs reduced the expression of target inflammation-associated genes (*Socs3*, *Tnf*, *Mtor*, and *Atf6*) (Stengel et al., 2020; Zhu et al., 2021; Wang et al., 2022; Zhou et al., 2022) in ethanol-treated mice, suggesting their involvement in enhancing the immune regulatory potential to fight ethanol-induced neuroinflammation. Considering that MSC-EVs could also elicit hepatoprotective effects against drug- or chemical-induced liver injury, these miRNAs may promote liver regeneration by modulating metabolic pathways and immune responses in hepatocytes (Cho et al., 2018), which could contribute to the observed effects of this study.

It is important to consider that sex is a significant biological factor influencing the neurobiology and pathological consequences associated with chronic alcohol consumption. Structurally, different brain areas are more affected in males or females, leading to sex-based differences in behavioral patterns associated with alcoholism (Maggioni et al., 2023). For example, males tend to experience higher rates of physical and behavioral problems, whereas females face a greater risk of developing physical and psychiatric comorbidities (Erol and Karpyak, 2015; Flores-Bonilla and Richardson, 2020). Moreover, we have previously demonstrated that female mice are more vulnerable to the neurotoxic effects of ethanol, exhibiting a stronger neuroinflammatory response than male mice (Alfonso-Loeches et al., 2013; Pascual et al., 2017a). In this context, this study uses female mice, despite the fact that neuroscience research typically favors male mice to avoid behavioral variations induced by the hormonal cycle.

We acknowledge certain limitations in the design and methodology of our study. In addition to the challenges of obtaining the required quantity of MSC-EVs, the chronic treatment and the high number of animals needed are primary concerns. Further studies are required to clarify the role of these miRNAs in ethanol-induced neuroinflammation and behavior alterations, potentially through the use of miRNA inhibitors or mimics. In this sense, exploring other functional miRNAs within MSCs-EVs and their target genes via cellular interference could further elucidate their roles in the effects of ethanol consumption. Finally, investigating additional regulatory molecular networks involved in MSC-EVs-mediated effects on ethanol-induced behavioral alterations may yield valuable insights.

Taken together, these novel results support the protective role of MSC-EVs in mitigating the neuroinflammatory immune response, cognitive dysfunction, and conditioned rewarding effects of cocaine induced by chronic alcohol consumption. Identifying specific miRNAs and their target genes related to the immune response could provide a deeper understanding of the mechanisms underlying the neurotoxicity associated with alcohol consumption.

## Additional files:

***Additional Table 1:*** *Nucleotide sequences of the primers used for qPCR of genes.*

***Additional Table 2:*** *Nucleotide sequences of the primers used for qPCR of microRNAs.*

***Additional Table 3:*** *Time spent (in seconds) in the non-associated compartment and associated compartment in both pre-conditioning and post-conditioning sessions in the conditioned place preference task.*

***Additional Table 4:*** *The total number of entries or crossings during the post-conditioning sessions in the conditioned place preference task.*

***Additional Table 5:*** *Summary of statistical results.*

Additional Table 5 Summary of statistical results

***Additional Figure 1:*** *Investigation time in both objects in the training and test sessions in the novel object recognition task.*

Additional Figure 1 Investigation time in both objects in the training and test sessions in the novel object recognition task.
Data presented as mean ± SEM, n=14-15 mice/group. ^**^P < 0.01, ^***^P < 0.001, *vs*. control mice; #*P* < 0.05, *vs*. MSC-EVs-treated mice. Two-way ANOVA with Bonferroni multiple comparisons test was performed.

***Additional Figure 2:*** *Protein–protein interaction for the predicted genes of miR-483-5p and miR-140-3p.*

Additional Figure 2 Protein–protein interaction for the predicted genes of miR-483-5p and miR-140-3p.

***Additional Figure 3:*** *Bar plot of the significant pathways enriched in the genes from the protein–protein interaction network derived from the target genes of miR-483-5p and miR-140-3p, enriched in the nervous system.*

Additional Figure 3 Bar plot of the significant pathways enriched in the genes from the protein – protein interaction network derived from the target genes of miR-483-5p and miR-140-3p, enriched in the nervous system.
The bar size indicates the number of genes involved in each pathway, and the color represents the FDR value.

## Data Availability

*The datasets generated and analyzed during the current study are available in the Zenodo repository: https://doi.org/10.5281/zenodo.13899491*.
