# Supplementary figures and images for: Role of miRNAs from mesenchymal stem cell–derived extracellular vesicles in neuroinflammation and behavioral impairments induced by chronic alcohol consumption in female mice
Source: Neural Regen Res. 2025 Jun 19;21(6):2370–9. doi: 10.4103/NRR.NRR-D-24-01260 (PMC13211783; doi:10.4103/NRR.NRR-D-24-01260)

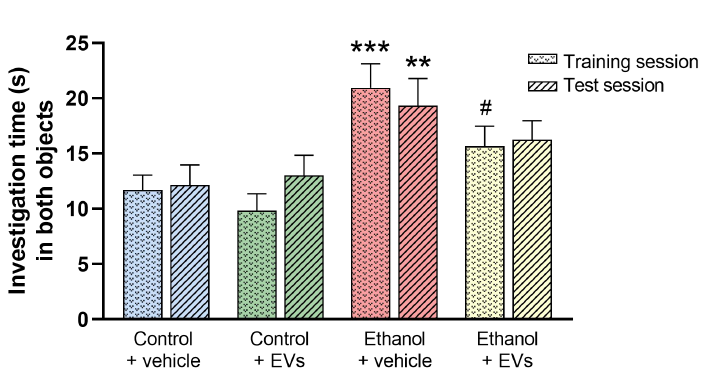

Supplement: Supplementary file 1 [file NRR-21-2370_Suppl1.tif]

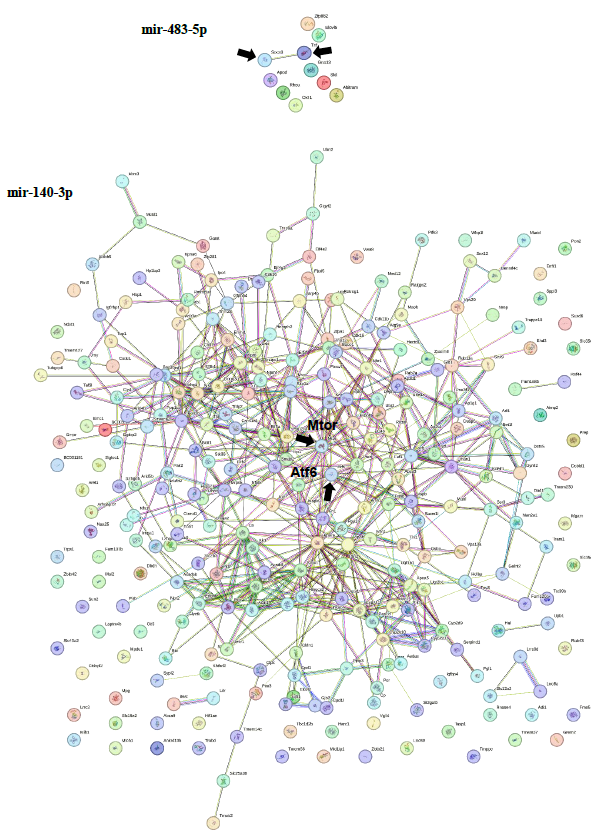

Supplement: Supplementary file 3 [file NRR-21-2370_Suppl2.tif]

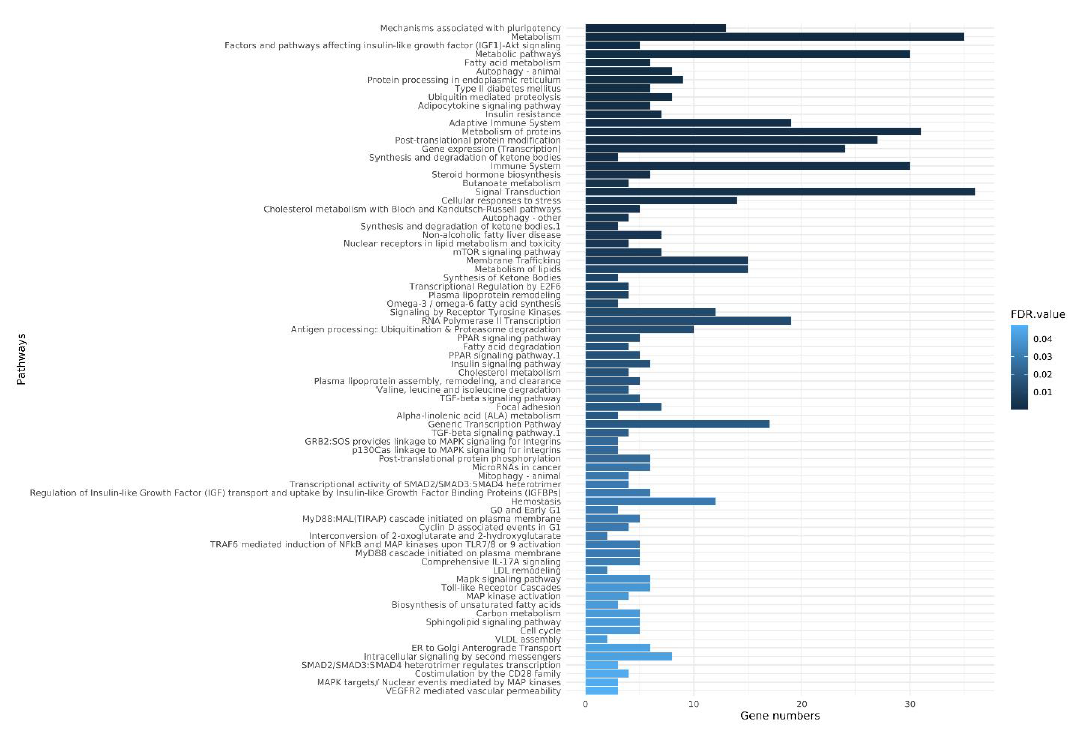

Supplement: Supplementary file 4 [file NRR-21-2370_Suppl3.tif]
