# Supplementary material for: Role of miRNAs from mesenchymal stem cell–derived extracellular vesicles in neuroinflammation and behavioral impairments induced by chronic alcohol consumption in female mice
Source: Neural Regen Res. 2025 Jun 19;21(6):2370–9. doi: 10.4103/NRR.NRR-D-24-01260 (PMC13211783; doi:10.4103/NRR.NRR-D-24-01260)
Supplement: Supplementary file 2 [file NRR-21-2370_Suppl1.pdf]

Additional Table 5 Summary of statistical results

Figure	Data	Comparison	Test	Statistics
**2A**	Activity concentration (kBq/cm^3)	Vehicle vs. EVs	Two-way ANOVA	$F_{(1, 18)} = 4.606$, $P < 0.05$
**2A**	Activity concentration (kBq/cm^3)	Ethanol vs. Control	Two-way ANOVA	$F_{(1, 18)} = 5.104$, $P < 0.05$
**2A**	Activity concentration (kBq/cm^3)	Interaction	Two-way ANOVA	$F_{(1, 18)} = 4.171$, $P > 0.05$
**2B**	Prefrontal cortex activity concentration (kBq/cm^3)	Vehicle vs. EVs	Two-way ANOVA	$F_{(1, 18)} = 5.845$, $P < 0.05$
**2B**	Prefrontal cortex activity concentration (kBq/cm^3)	Ethanol vs. Control	Two-way ANOVA	$F_{(1, 18)} = 6.338$, $P < 0.05$
**2B**	Prefrontal cortex activity concentration (kBq/cm^3)	Interaction	Two-way ANOVA	$F_{(1, 18)} = 5.424$, $P < 0.05$
**2B**	Hippocampus dorsal activity concentration (kBq/cm^3)	Vehicle vs. EVs	Two-way ANOVA	$F_{(1, 18)} = 6.077$, $P < 0.05$
**2B**	Hippocampus dorsal activity concentration (kBq/cm^3)	Ethanol vs. Control	Two-way ANOVA	$F_{(1, 18)} = 6.570$, $P < 0.05$
**2B**	Hippocampus dorsal activity concentration (kBq/cm^3)	Interaction	Two-way ANOVA	$F_{(1, 18)} = 5.446$, $P < 0.05$
**2B**	Hippocampus ventral activity concentration (kBq/cm^3)	Vehicle vs. EVs	Two-way ANOVA	$F_{(1, 18)} = 4.500$, $P < 0.05$
**2B**	Hippocampus ventral activity concentration (kBq/cm^3)	Ethanol vs. Control	Two-way ANOVA	$F_{(1, 18)} = 8.072$, $P < 0.05$
**2B**	Hippocampus ventral activity concentration (kBq/cm^3)	Interaction	Two-way ANOVA	$F_{(1, 18)} = 5.092$, $P < 0.05$
**2B**	Striatum dorsal activity concentration (kBq/cm^3)	Vehicle vs. EVs	Two-way ANOVA	$F_{(1, 18)} = 4.356$, $P > 0.05$
**2B**	Striatum dorsal activity concentration (kBq/cm^3)	Ethanol vs. Control	Two-way ANOVA	$F_{(1, 18)} = 8.041$, $P < 0.05$
**2B**	Striatum dorsal activity concentration (kBq/cm^3)	Interaction	Two-way ANOVA	$F_{(1, 18)} = 4.002$, $P > 0.05$
**2B**	Striatum ventral activity concentration (kBq/cm^3)	Vehicle vs. EVs	Two-way ANOVA	$F_{(1, 18)} = 5.811$, $P < 0.05$
**2B**	Striatum ventral activity concentration (kBq/cm^3)	Ethanol vs. Control	Two-way ANOVA	$F_{(1, 18)} = 6.312$, $P < 0.05$
**2B**	Striatum ventral activity concentration (kBq/cm^3)	Ethanol vs. Control	Two-way ANOVA	$F_{(1, 18)} = 5.450$, $P < 0.05$
**2C**	Whole brain volume (mm^3)	Vehicle vs. EVs	Two-way ANOVA	$F_{(1, 19)} = 0.08415$, $P > 0.05$
**2C**	Whole brain volume (mm^3)	Ethanol vs. Control	Two-way ANOVA	$F_{(1, 19)} = 18.04$, $P < 0.001$
**2C**	Whole brain volume (mm^3)	Interaction	Two-way ANOVA	$F_{(1, 19)} = 1.494$, $P > 0.05$
**2C**	Hippocampal thickness (mm) height	Vehicle vs. EVs	Two-way ANOVA	$F_{(1, 19)} = 2.554$, $P > 0.05$
**2C**	Hippocampal thickness (mm) height	Ethanol vs. Control	Two-way ANOVA	$F_{(1, 19)} = 2.598$, $P > 0.05$
**2C**	Hippocampal thickness (mm) height	Interaction	Two-way ANOVA	$F_{(1, 19)} = 0.4726$, $P > 0.05$
**2C**	Hippocampal thickness (mm) widht	Vehicle vs. EVs	Two-way ANOVA	$F_{(1, 19)} = 4.314$, $P < 0.05$
**2C**	Hippocampal thickness (mm) widht	Ethanol vs. Control	Two-way ANOVA	$F_{(1, 19)} = 4.588$, $P < 0.05$
**2C**	Hippocampal thickness (mm) widht	Interaction	Two-way ANOVA	$F_{(1, 19)} = 2.762$, $P > 0.05$
**2C**	Cortical thickness (mm) cleft	Vehicle vs. EVs	Two-way ANOVA	$F_{(1, 19)} = 4.585$, $P < 0.05$
**2C**	Cortical thickness (mm) cleft	Ethanol vs. Control	Two-way ANOVA	$F_{(1, 19)} = 5.698$, $P < 0.05$
**2C**	Cortical thickness (mm) cleft	Interaction	Two-way ANOVA	$F_{(1, 19)} = 3.187$, $P > 0.05$
**2C**	Cortical thickness (mm) lateral	Vehicle vs. EVs	Two-way ANOVA	$F_{(1, 19)} = 4.301$, $P < 0.05$
**2C**	Cortical thickness (mm) lateral	Ethanol vs. Control	Two-way ANOVA	$F_{(1, 19)} = 5.293$, $P < 0.05$
**2C**	Cortical thickness (mm) lateral	Interaction	Two-way ANOVA	$F_{(1, 19)} = 2.854$, $P > 0.05$
**3A**	Prefrontal cortex Il1b mRNA fold change	Vehicle vs. EVs	Two-way ANOVA	$F_{(1, 32)} = 5.996$, $P < 0.05$
**3A**	Prefrontal cortex Il1b mRNA fold change	Ethanol vs. Control	Two-way ANOVA	$F_{(1, 32)} = 7.146$, $P < 0.05$
**3A**	Prefrontal cortex Il1b mRNA	Interaction	Two-way	$F_{(1, 32)} = 5.214$, $P < 0.05$

	fold change		ANOVA	
**3A**	Prefrontal cortex Il6 mRNA fold change	Vehicle vs. EVs	Two-way ANOVA	$F_{(1, 32)} = 1.045$, $P > 0.05$
**3A**	Prefrontal cortex Il6 mRNA fold change	Ethanol vs. Control	Two-way ANOVA	$F_{(1, 32)} = 8.313$, $P < 0.01$
**3A**	Prefrontal cortex Il6 mRNA fold change	Interaction	Two-way ANOVA	$F_{(1, 32)} = 9.885$, $P < 0.01$
**3A**	Prefrontal cortex Ccl2 mRNA fold change	Vehicle vs. EVs	Two-way ANOVA	$F_{(1, 32)} = 3.337$, $P > 0.05$
**3A**	Prefrontal cortex Ccl2 mRNA fold change	Ethanol vs. Control	Two-way ANOVA	$F_{(1, 32)} = 8.207$, $P < 0.01$
**3A**	Prefrontal cortex Ccl2 mRNA fold change	Interaction	Two-way ANOVA	$F_{(1, 32)} = 5.916$, $P < 0.05$
**3A**	Prefrontal cortex Ccl3 mRNA fold change	Vehicle vs. EVs	Two-way ANOVA	$F_{(1, 32)} = 3.396$, $P > 0.05$
**3A**	Prefrontal cortex Ccl3 mRNA fold change	Ethanol vs. Control	Two-way ANOVA	$F_{(1, 32)} = 2.585$, $P > 0.05$
**3A**	Prefrontal cortex Ccl3 mRNA fold change	Interaction	Two-way ANOVA	$F_{(1, 32)} = 6.042$, $P < 0.05$
**3A**	Prefrontal cortex Cxcl1 mRNA fold change	Vehicle vs. EVs	Two-way ANOVA	$F_{(1, 32)} = 0.03905$, $P > 0.05$
**3A**	Prefrontal cortex Cxcl1 mRNA fold change	Ethanol vs. Control	Two-way ANOVA	$F_{(1, 32)} = 0.04337$, $P > 0.05$
**3A**	Prefrontal cortex Cxcl1 mRNA fold change	Interaction	Two-way ANOVA	$F_{(1, 32)} = 0.05340$, $P > 0.05$
**3A**	Prefrontal cortex Nos2 mRNA fold change	Vehicle vs. EVs	Two-way ANOVA	$F_{(1, 32)} = 13.19$, $P = 0.001$
**3A**	Prefrontal cortex Nos2 mRNA fold change	Ethanol vs. Control	Two-way ANOVA	$F_{(1, 32)} = 4.825$, $P < 0.05$
**3A**	Prefrontal cortex Nos2 mRNA fold change	Interaction	Two-way ANOVA	$F_{(1, 32)} = 5.523$, $P < 0.05$
**3A**	Prefrontal cortex Cos2 mRNA fold change	Vehicle vs. EVs	Two-way ANOVA	$F_{(1, 32)} = 0.007597$, $P > 0.05$
**3A**	Prefrontal cortex Cos2 mRNA fold change	Ethanol vs. Control	Two-way ANOVA	$F_{(1, 32)} = 0.2988$, $P > 0.05$
**3A**	Prefrontal cortex Cos2 mRNA fold change	Interaction	Two-way ANOVA	$F_{(1, 32)} = 0.001886$, $P > 0.05$
**3A**	Striatum Il1b mRNA fold change	Vehicle vs. EVs	Two-way ANOVA	$F_{(1, 32)} = 11.13$, $P < 0.01$
**3A**	Striatum Il1b mRNA fold change	Ethanol vs. Control	Two-way ANOVA	$F_{(1, 32)} = 8.007$, $P < 0.01$
**3A**	Striatum Il1b mRNA fold change	Interaction	Two-way ANOVA	$F_{(1, 32)} = 10.99$, $P < 0.01$
**3A**	Striatum Il6 mRNA fold change	Vehicle vs. EVs	Two-way ANOVA	$F_{(1, 32)} = 11.58$, $P < 0.01$
**3A**	Striatum Il6 mRNA fold change	Ethanol vs. Control	Two-way ANOVA	$F_{(1, 32)} = 4.203$, $P < 0.05$
**3A**	Striatum Il6 mRNA fold change	Interaction	Two-way ANOVA	$F_{(1, 32)} = 15.62$, $P < 0.001$
**3A**	Striatum Ccl2 mRNA fold change	Vehicle vs. EVs	Two-way ANOVA	$F_{(1, 32)} = 12.28$, $P < 0.01$
**3A**	Striatum Ccl2 mRNA fold change	Ethanol vs. Control	Two-way ANOVA	$F_{(1, 32)} = 1.782$, $P > 0.05$
**3A**	Striatum Ccl2 mRNA fold change	Interaction	Two-way ANOVA	$F_{(1, 32)} = 9.036$, $P < 0.01$
**3A**	Striatum Ccl3 mRNA fold change	Vehicle vs. EVs	Two-way ANOVA	$F_{(1, 32)} = 15.12$, $P < 0.001$
**3A**	Striatum Ccl3 mRNA fold change	Ethanol vs. Control	Two-way ANOVA	$F_{(1, 32)} = 10.02$, $P < 0.01$
**3A**	Striatum Ccl3 mRNA fold change	Interaction	Two-way ANOVA	$F_{(1, 32)} = 7.983$, $P < 0.01$
**3A**	Striatum Cxcl1 mRNA fold change	Vehicle vs. EVs	Two-way ANOVA	$F_{(1, 32)} = 0.8181$, $P > 0.05$
**3A**	Striatum Cxcl1 mRNA fold change	Ethanol vs. Control	Two-way ANOVA	$F_{(1, 32)} = 0.04463$, $P > 0.05$
**3A**	Striatum Cxcl1 mRNA fold change	Interaction	Two-way ANOVA	$F_{(1, 32)} = 1.411$, $P > 0.05$
**3A**	Striatum Nos2 mRNA fold change	Vehicle vs. EVs	Two-way ANOVA	$F_{(1, 32)} = 9.274$, $P < 0.01$
**3A**	Striatum Nos2 mRNA fold change	Ethanol vs. Control	Two-way ANOVA	$F_{(1, 32)} = 2.906$, $P > 0.05$
**3A**	Striatum Nos2 mRNA fold change	Interaction	Two-way ANOVA	$F_{(1, 32)} = 4.680$, $P < 0.05$

**3A**	Striatum Cos2 mRNA fold change	Vehicle vs. EVs	Two-way ANOVA	$F_{(1, 32)} = 0.3210$, $P > 0.05$
**3A**	Striatum Cos2 mRNA fold change	Ethanol vs. Control	Two-way ANOVA	$F_{(1, 32)} = 0.01270$, $P > 0.05$
**3A**	Striatum Cos2 mRNA fold change	Interaction	Two-way ANOVA	$F_{(1, 32)} = 0.9277$, $P > 0.05$
**3A**	Hippocampus Il1b mRNA fold change	Vehicle vs. EVs	Two-way ANOVA	$F_{(1, 32)} = 4.224$, $P > 0.05$
**3A**	Hippocampus Il1b mRNA fold change	Ethanol vs. Control	Two-way ANOVA	$F_{(1, 32)} = 25.90$, $P < 0.0001$
**3A**	Hippocampus Il1b mRNA fold change	Interaction	Two-way ANOVA	$F_{(1, 32)} = 29.81$, $P < 0.0001$
**3A**	Hippocampus Il6 mRNA fold change	Vehicle vs. EVs	Two-way ANOVA	$F_{(1, 32)} = 2.700$, $P > 0.05$
**3A**	Hippocampus Il6 mRNA fold change	Ethanol vs. Control	Two-way ANOVA	$F_{(1, 32)} = 5.112$, $P < 0.05$
**3A**	Hippocampus Il6 mRNA fold change	Interaction	Two-way ANOVA	$F_{(1, 32)} = 8.124$, $P < 0.01$
**3A**	Hippocampus Ccl2 mRNA fold change	Vehicle vs. EVs	Two-way ANOVA	$F_{(1, 32)} = 5.753$, $P < 0.05$
**3A**	Hippocampus Ccl2 mRNA fold change	Ethanol vs. Control	Two-way ANOVA	$F_{(1, 32)} = 15.67$, $P < 0.001$
**3A**	Hippocampus Ccl2 mRNA fold change	Interaction	Two-way ANOVA	$F_{(1, 32)} = 8.422$, $P < 0.01$
**3A**	Hippocampus Ccl3 mRNA fold change	Vehicle vs. EVs	Two-way ANOVA	$F_{(1, 32)} = 3.137$, $P > 0.05$
**3A**	Hippocampus Ccl3 mRNA fold change	Ethanol vs. Control	Two-way ANOVA	$F_{(1, 32)} = 2.22$, $P < 0.01$
**3A**	Hippocampus Ccl3 mRNA fold change	Interaction	Two-way ANOVA	$F_{(1, 32)} = 10.89$, $P < 0.01$
**3A**	Hippocampus Cxcl1 mRNA fold change	Vehicle vs. EVs	Two-way ANOVA	$F_{(1, 32)} = 0.2051$, $P > 0.05$
**3A**	Hippocampus Cxcl1 mRNA fold change	Ethanol vs. Control	Two-way ANOVA	$F_{(1, 32)} = 0.003054$, $P > 0.05$
**3A**	Hippocampus Cxcl1 mRNA fold change	Interaction	Two-way ANOVA	$F_{(1, 32)} = 0.1128$, $P > 0.05$
**3A**	Hippocampus Nos2 mRNA fold change	Vehicle vs. EVs	Two-way ANOVA	$F_{(1, 32)} = 6.297$, $P < 0.05$
**3A**	Hippocampus Nos2 mRNA fold change	Ethanol vs. Control	Two-way ANOVA	$F_{(1, 32)} = 7.291$, $P < 0.05$
**3A**	Hippocampus Nos2 mRNA fold change	Interaction	Two-way ANOVA	$F_{(1, 32)} = 4.780$, $P < 0.05$
**3A**	Hippocampus Cos2 mRNA fold change	Vehicle vs. EVs	Two-way ANOVA	$F_{(1, 32)} = 0.0006785$, $P > 0.05$
**3A**	Hippocampus Cos2 mRNA fold change	Ethanol vs. Control	Two-way ANOVA	$F_{(1, 32)} = 0.6115$, $P > 0.05$
**3A**	Hippocampus Cos2 mRNA fold change	Interaction	Two-way ANOVA	$F_{(1, 32)} = 0.003160$, $P > 0.05$
**3D**	Radiant efficiency (arbitrary units)	Vehicle vs. EVs	Two-way ANOVA	$F_{(1, 17)} = 6.880$, $P < 0.05$
**3D**	Radiant efficiency (arbitrary units)	Ethanol vs. Control	Two-way ANOVA	$F_{(1, 17)} = 8.335$, $P < 0.05$
**3D**	Radiant efficiency (arbitrary units)	Interaction	Two-way ANOVA	$F_{(1, 17)} = 7.249$, $P < 0.05$
**4C**	Discrimination index (%)	Vehicle vs. EVs	Two-way ANOVA	$F_{(1, 54)} = 3.922$, $P > 0.05$
**4C**	Discrimination index (%)	Ethanol vs. Control	Two-way ANOVA	$F_{(1, 54)} = 7.207$, $P = 0.01$
**4C**	Discrimination index (%)	Interaction	Two-way ANOVA	$F_{(1, 54)} = 4.505$, $P < 0.05$
**4D**	Time (s) to enter in the dark compartment (training day)	Control + vehicle vs. Control + EVs	Mann-Whitney U test	$U = 76$, $P > 0.05$
**4D**	Time (s) to enter in the dark compartment (training day)	Control + vehicle vs. Ethanol + vehicle	Mann-Whitney U test	$U = 55$, $P < 0.05$
**4D**	Time (s) to enter in the dark compartment (training day)	Control + vehicle vs. Ethanol + EVs	Mann-Whitney U test	$U = 76$, $P > 0.05$
**4D**	Time (s) to enter in the dark compartment (training day)	Control + EVs vs. Ethanol + vehicle	Mann-Whitney U	$U = 37.5$, $P < 0.01$

			test	
**4D**	Time (s) to enter in the dark compartment (training day)	Control + EVs vs. Ethanol + EVs	Mann-Whitney *U* test	$U = 106.5$, $P > 0.05$
**4D**	Time (s) to enter in the dark compartment (training day)	Ethanol + vehicle vs. Ethanol + EVs	Mann-Whitney *U* test	$U = 27.5$, $P < 0.001$
**4D**	Time (s) to enter in the dark compartment (test day)	Control + vehicle vs. Control + EVs	Mann-Whitney *U* test	$U = 70$, $P > 0.05$
**4D**	Time (s) to enter in the dark compartment (test day)	Control + vehicle vs. Ethanol + vehicle	Mann-Whitney *U* test	$U = 37$, $P < 0.01$
**4D**	Time (s) to enter in the dark compartment (test day)	Control + vehicle vs. Ethanol + EVs	Mann-Whitney *U* test	$U = 65$, $P < 0.05$
**4D**	Time (s) to enter in the dark compartment (test day)	Control + EVs vs. Ethanol + vehicle	Mann-Whitney *U* test	$U = 31$, $P = 0.01$
**4D**	Time (s) to enter in the dark compartment (test day)	Control + EVs vs. Ethanol + EVs	Mann-Whitney *U* test	$U = 55$, $P > 0.05$
**4D**	Time (s) to enter in the dark compartment (test day)	Ethanol + vehicle vs. Ethanol + EVs	Mann-Whitney *U* test	$U = 60$, $P < 0.05$
**4E**	Time in drug-paired compartment (Pre-C vs. Post-C)	Control	*t*-test	$t = -2.249$, $P < 0.05$
**4E**	Time in drug-paired compartment (Pre-C vs. Post-C)	EVs	*t*-test	$t = -2.409$, $P < 0.05$
**4E**	Time in drug-paired compartment (Pre-C vs. Post-C)	Ethanol	*t*-test	$t = -0.387$, $P > 0.05$
**4E**	Time in drug-paired compartment (Pre-C vs. Post-C)	Ethanol + EVs	*t*-test	$t = -2.258$, $P < 0.05$
**5D**	Hippocampus miR-483-5p expression fold change	Vehicle vs. EVs	Two-way ANOVA	$F_{(1, 22)} = 12.33$, $P < 0.01$
**5D**	Hippocampus miR-483-5p expression fold change	Ethanol vs. Control	Two-way ANOVA	$F_{(1, 22)} = 0.8074$, $P > 0.05$
**5D**	Hippocampus miR-483-5p expression fold change	Interaction	Two-way ANOVA	$F_{(1, 22)} = 2.290$, $P > 0.05$
**5D**	Hippocampus miR-140-5p expression fold change	Vehicle vs. EVs	Two-way ANOVA	$F_{(1, 22)} = 2.468$, $P > 0.05$
**5D**	Hippocampus miR-140-5p expression fold change	Ethanol vs. Control	Two-way ANOVA	$F_{(1, 22)} = 5.232$, $P < 0.05$
**5D**	Hippocampus miR-140-5p expression fold change	Interaction	Two-way ANOVA	$F_{(1, 22)} = 0.002262$, $P > 0.05$
**5D**	Prefrontal cortex miR-483-5p expression fold change	Vehicle vs. EVs	Two-way ANOVA	$F_{(1, 22)} = 0.001728$, $P > 0.05$
**5D**	Prefrontal cortex miR-483-5p expression fold change	Ethanol vs. Control	Two-way ANOVA	$F_{(1, 22)} = 2.503$, $P > 0.05$
**5D**	Prefrontal cortex miR-483-5p expression fold change	Interaction	Two-way ANOVA	$F_{(1, 22)} = 0.07651$, $P > 0.05$
**5D**	Prefrontal cortex miR-140-5p expression fold change	Vehicle vs. EVs	Two-way ANOVA	$F_{(1, 22)} = 15.33$, $P = 0.001$
**5D**	Prefrontal cortex miR-140-5p expression fold change	Ethanol vs. Control	Two-way ANOVA	$F_{(1, 22)} = 2.008$, $P > 0.05$
**5D**	Prefrontal cortex miR-140-5p expression fold change	Interaction	Two-way ANOVA	$F_{(1, 22)} = 0.0003618$, $P > 0.05$
**5E**	Hippocampus Socs3 mRNA expression fold change	Vehicle vs. EVs	Two-way ANOVA	$F_{(1, 22)} = 2.790$, $P > 0.05$
**5E**	Hippocampus Socs3 mRNA expression fold change	Ethanol vs. Control	Two-way ANOVA	$F_{(1, 22)} = 1.06$, $P < 0.01$
**5E**	Hippocampus Socs3 mRNA expression fold change	Interaction	Two-way ANOVA	$F_{(1, 22)} = 8.703$, $P < 0.01$
**5E**	Hippocampus Tnf mRNA expressionn fold change	Vehicle vs. EVs	Two-way ANOVA	$F_{(1, 22)} = 2.857$, $P > 0.05$
**5E**	Hippocampus Tnf mRNA expression fold change	Ethanol vs. Control	Two-way ANOVA	$F_{(1, 22)} = 6.102$, $P < 0.05$
**5E**	Hippocampus Tnf mRNA expression fold change	Interaction	Two-way ANOVA	$F_{(1, 22)} = 11.76$, $P < 0.01$

**5E**	Prefrontal cortex Mtor mRNA expression	Vehicle vs. EVs	Two-way ANOVA	$F_{(1, 22)} = 25.22$, $P < 0.0001$
**5E**	Prefrontal cortex Mtor mRNA expression	Ethanol vs. Control	Two-way ANOVA	$F_{(1, 22)} = 0.8237$, $P > 0.05$
**5E**	Prefrontal cortex Mtor mRNA expression	Interaction	Two-way ANOVA	$F_{(1, 22)} = 1.799$, $P > 0.05$
**5E**	Prefrontal cortex Atf6 mRNA expression	Vehicle vs. EVs	Two-way ANOVA	$F_{(1, 22)} = 9.439$, $P < 0.01$
**5E**	Prefrontal cortex Atf6 mRNA expression	Ethanol vs. Control	Two-way ANOVA	$F_{(1, 22)} = 6.588$, $P < 0.05$
**5E**	Prefrontal cortex Atf6 mRNA expression	Interaction	Two-way ANOVA	$F_{(1, 22)} = 3.268$, $P > 0.05$
**Additional Fig. 1**	Investigation time in the training day	Vehicle vs. EVs	Two-way ANOVA	$F_{(1, 54)} = 4.538$, $P < 0.05$
**Additional Fig. 1**	Investigation time in the training day	Ethanol vs. Control	Two-way ANOVA	$F_{(1, 54)} = 19.989$, $P < 0.001$
**Additional Fig. 1**	Investigation time in the training day	Interaction	Two-way ANOVA	$F_{(1, 54)} = 1.042$, $P > 0.05$
**Additional Fig. 1**	Investigation time in the test day	Vehicle vs. EVs	Two-way ANOVA	$F_{(1, 54)} = 0.349$, $P > 0.05$
**Additional Fig. 1**	Investigation time in the test day	Ethanol vs. Control	Two-way ANOVA	$F_{(1, 54)} = 7.62$, $P < 0.01$
**Additional Fig. 1**	Investigation time in the test day	Interaction	Two-way ANOVA	$F_{(1, 54)} = 1.106$, $P > 0.05$
**Additional Table 3**	Time associated compartment vs non-associated in the pre-C vs post-C	Test	Two-way ANOVA	$F_{(1, 98)} = 3.182$, $P > 0.05$
**Additional Table 3**	Time associated compartment vs non-associated in the pre-C vs post-C	Test vs. EVs	Two-way ANOVA	$F_{(1, 98)} = 0.089$, $P > 0.05$
**Additional Table 3**	Time associated compartment vs non-associated in the pre-C vs post-C	Test vs. Ethanol	Two-way ANOVA	$F_{(1, 98)} = 0.502$, $P > 0.05$
**Additional Table 3**	Time associated compartment vs non-associated in the pre-C vs post-C	Test vs. Compartment	Two-way ANOVA	$F_{(1, 98)} = 25.803$, $P < 0.05$ **(1)**
**Additional Table 3**	Time associated compartment vs non-associated in the pre-C vs post-C	Test vs. EVs vs. Ethanol	Two-way ANOVA	$F_{(1, 98)} = 0.007$, $P > 0.05$
**Additional Table 3**	Time associated compartment vs non-associated in the pre-C vs post-C	Test vs. EVs vs. Compartment	Two-way ANOVA	$F_{(1, 98)} = 0.207$, $P > 0.05$
**Additional Table 3**	Time associated compartment vs non-associated in the pre-C vs post-C	Test vs. Ethanol vs. Compartment	Two-way ANOVA	$F_{(1, 98)} = 0.262$, $P > 0.05$
**Additional Table 3**	Time associated compartment vs non-associated in the pre-C vs post-C	Test vs. EVs vs. Ethanol vs. Compartment	Two-way ANOVA	$F_{(1, 98)} = 0.036$, $P > 0.05$
**Additional Table 3**	Time associated compartment vs non-associated in the pre-C vs post-C	Ethanol	Two-way ANOVA	$F_{(1, 98)} = 0.345$, $P > 0.05$
**Additional Table 3**	Time associated compartment vs non-associated in the pre-C vs post-C	EVs	Two-way ANOVA	$F_{(1, 98)} = 0.112$, $P > 0.05$
**Additional Table 3**	Time associated compartment vs non-associated in the pre-C vs post-C	Compartment	Two-way ANOVA	$F_{(1, 98)} = 10.36$, $P < 0.05$ **(2)**
**Additional Table 3**	Time associated compartment vs non-associated in the pre-C vs post-C	EVs vs. Ethanol	Two-way ANOVA	$F_{(1, 98)} = 0.141$, $P > 0.05$
**Additional Table 4**	Total crossings in the pre-C	Vehicle vs. EVs	Two-way ANOVA	$F_{(1, 52)} = 0.002$, $P > 0.05$
**Additional Table 4**	Total crossings in the pre-C	Ethanol vs. Control	Two-way ANOVA	$F_{(1, 52)} = 0.711$, $P > 0.05$
**Additional Table 4**	Total crossings in the pre-C	Interaction	Two-way ANOVA	$F_{(1, 52)} = 2.727$, $P > 0.05$
**Additional Table 4**	Total crossings in the post-C	Vehicle vs. EVs	Two-way ANOVA	$F_{(1, 52)} = 0.097$, $P > 0.05$
**Additional Table 4**	Total crossings in the post-C	Ethanol vs. Control	Two-way ANOVA	$F_{(1, 52)} = 0.001$, $P > 0.05$
**Additional Table 4**	Total crossings in the post-C	Interaction	Two-way ANOVA	$F_{(1, 52)} = 0.009$, $P > 0.05$

ANOVA: Analysis of variance; EVs: extracellular vesicles; post-C: post-conditioning; pre-C: pre-conditioning.
